# Automatic ovarian tumors recognition system based on ensemble convolutional neural network with ultrasound imaging

**DOI:** 10.1186/s12911-022-02047-6

**Published:** 2022-11-17

**Authors:** Shih-Tien Hsu, Yu-Jie Su, Chian-Huei Hung, Ming-Jer Chen, Chien-Hsing Lu, Chih-En Kuo

**Affiliations:** 1 Department of Obstetrics, Gynecology and Women’s Health, Taichung Veterans General Hospital, No. 1650 Sec. 4 Taiwan Blvd. Xitun Dist., Taichung, 407 Taiwan; 2 Master’s Program of Biomedical Infomatics and Biomedical Engineering, Feng Chia University, No. 100 Wenhua Rd. Xitun Dist., Taichung, 407 Taiwan; 3 Department of Applied Mathematics, National Chung Hsing University, No. 145, Xingda Rd., South Dist., Taichung, 402 Taiwan

**Keywords:** Convolution neural network, Ovarian tumor, Ultrasound diagnosis, Grad-CAM, Ensemble learning

## Abstract

**Background:**

Upon the discovery of ovarian cysts, obstetricians, gynecologists, and ultrasound examiners must address the common clinical challenge of distinguishing between benign and malignant ovarian tumors. Numerous types of ovarian tumors exist, many of which exhibit similar characteristics that increase the ambiguity in clinical diagnosis. Using deep learning technology, we aimed to develop a method that rapidly and accurately assists the different diagnosis of ovarian tumors in ultrasound images.

**Methods:**

Based on deep learning method, we used ten well-known convolutional neural network models (e.g., Alexnet, GoogleNet, and ResNet) for training of transfer learning. To ensure method stability and robustness, we repeated the random sampling of the training and validation data ten times. The mean of the ten test results was set as the final assessment data. After the training process was completed, the three models with the highest ratio of calculation accuracy to time required for classification were used for ensemble learning pertaining. Finally, the interpretation results of the ensemble classifier were used as the final results. We also applied ensemble gradient-weighted class activation mapping (Grad-CAM) technology to visualize the decision-making results of the models.

**Results:**

The highest mean accuracy, mean sensitivity, and mean specificity of ten single CNN models were 90.51 ± 4.36%, 89.77 ± 4.16%, and 92.00 ± 5.95%, respectively. The mean accuracy, mean sensitivity, and mean specificity of the ensemble classifier method were 92.15 ± 2.84%, 91.37 ± 3.60%, and 92.92 ± 4.00%, respectively. The performance of the ensemble classifier is better than that of a single classifier in three evaluation metrics. Moreover, the standard deviation is also better which means the ensemble classifier is more stable and robust.

**Conclusion:**

From the comprehensive perspective of data quantity, data diversity, robustness of validation strategy, and overall accuracy, the proposed method outperformed the methods used in previous studies. In future studies, we will continue to increase the number of authenticated images and apply our proposed method in clinical settings to increase its robustness and reliability.

## Background

Ovarian and adnexal tumors are common gynecological problems that can affect women of all ages. Premenopausal women develop follicular and corpus luteum cysts that are less than three cm in size every month. Postmenopausal women still have a 13 to 16% chance of developing cysts [1]. Therefore, in clinical practice, pelvic ultrasound exams for women are likely to reveal ovarian and adnexal tumors, which require assessment, tracking, and treatment [2]. Most ovarian cysts are physiological and particularly common among premenopausal women, and they tend to disappear after a period of time. Only a small percentage of ovarian cysts are non-physiological, and such cysts will persist. Gynecologist can perform an abdominal or vaginal ultrasound to distinguish between benign and malignant cysts. Benign ovarian tumor often can be followed without interval change. Surgery only performed if benign tumors continue to grow or persist, torsion and rupture may occur. However, malignant tumors require immediate cytoreductive surgery followed by chemotherapy to reduce subsequent mortality. Ovarian cancer is the deadliest cancer of the female reproductive system. In 2012, approximately 240,000 women worldwide were diagnosed with ovarian cancer, and 150,000 died from ovarian cancer [3]. According to United States Cancer Statistics, 53% of patients with ovarian cancer die within 5 years of diagnosis, and the mortality rate of such patients can increase because of diagnostic delay and late-stage diagnosis [4].

Upon the discovery of ovarian cysts, obstetricians, gynecologists, and ultrasound examiners must address the common clinical challenge of distinguishing between benign and malignant ovarian tumors. Numerous types of ovarian tumors exist, many of which exhibit similar characteristics that increase the ambiguity in clinical diagnosis. An ambiguous diagnosis can increase the difficulty of making subsequent treatment decisions and cause more anxiety for a patient. For example, debulking surgery is a common surgical method for treating malignant tumors, and it involves large abdominal incisions. Benign ovarian tumors can be tracked or treated with minimally invasive surgery. An inaccurate diagnosis affects not only a patients’ wounds but also their prognosis.

At present, ultrasound evaluation methods for malignant ovarian tumors are primarily based on the Simple Rules published by the International Ovarian Tumor Analysis group. The Simple Rules for predicting a benign tumor (B features) include the following ultrasound features: unilocular cyst (B1), solid component measuring < 7 mm in diameter (B2), presence of acoustic shadows (B3), smooth multilocular tumor with maximum diameter being < 10 cm (B4), and absence of detectable color Doppler signal (B5). The Simple Rules for predicting a malignant tumor (M features) are as follows: irregular solid tumor (M1), ascites (M2), papillary structures (M3), irregular multilocular mass measuring > 10 cm in diameter (M4), and strong color Doppler signal (M5). When the Simple Rules are applied, tumors that exhibit only B features and those that only exhibit M features are classified as benign and malignant tumors, respectively. For tumors that exhibit none of the aforementioned features or both benign and malignant features, the Simple Rules are ineffective for achieving a clear differentiation. In 2008, Timmerman et al. applied the Simple Rules to manually interpret and classify 1233 tumor images, and 76% of the tumor images were recognizable with a sensitivity of 95% (106/112), a specificity of 91% (249/274) [5]. The difficulty and inconsistency of image interpretation can prevent physicians from identifying the appropriate treatment. Risk of malignancy index (RMI) is another popular approach to discriminate between benign and malignant adnexal tumors based on menopausal status, a transvaginal ultrasound score, and serum cancer antigen 125 (CA 125) level. The sensitivity was 85% and the specificity was 97%. The limitation is the need of another information [6]. Besides, the IOTA ADNEX model was also developed for risk estimates and applied as a next step in order to determine the risk of malignancy if ovarian malignancy was suspected [7]. Consequently, physicians can only wait until or after a surgery to know if they made the right decision. Consistency in image interpretation is related to the judgement and clinical experience of each physician.

Numerous studies have reported the successful application of deep learning technology in clinical settings for supporting image interpretation. Deep learning has been applied to ultrasound, X-ray, magnetic resonance imaging, computed tomography, and other images for image classification, object detection, and semantic segmentation [8–17]. Deep learning provides the advantage of learning discriminative features, which are implemented through repeated learning and correction to identify hidden rules and patterns in images, thereby improving the accuracy of classification. In recent years, some studies have also proposed the use of deep learning technology to diagnose ovarian tumors from various medical images [18, 19], including magnetic resonance imaging [20, 21], computed tomography [22], histopathological image [23], and ultrasound [24–27]. Ultrasonography is the most basic and commonly used way to distinguish benign and malignant ovarian tumors in clinical practice. Therefore, we aimed to develop a system that automates the characterization of ultrasound images of ovarian tumors; this is achieved by applying deep learning technology to help physicians improve their image interpretation accuracy and reduce the time required for interpretation. The results obtained through the system can serve as clinical references and reduce the risk of clinical misdiagnosis. To test the stability and robustness of the system, we selected ten well-known pre-trained models and compared the recognition accuracy of ovarian ultrasound images at the same conditions, and we repeated the random sampling of the training and validation data ten times for model training and validation, respectively. The mean of ten test results was used to evaluate the proposed method. In addition, the decision-making visualization technology of the proposed model enables physicians to understand the foundation of the system’s diagnostic results, such that they can assess the reliability of these system-derived results to prevent misdiagnoses.

## Methods

### Database

The datasets used for method development were collected from 587 patients at the Department of Obstetrics Gynecology and Women’s Health, Taichung Veterans General Hospital, Taiwan. These data collections were approved by the internal review board of Taichung Veterans General Hospital (IRB no. CE20356A). Informed consent obtained from all the participants included in the study. Two scanning techniques, abdominal ultrasound and vaginal ultrasound, were both included. In addition, the database does not contain color Doppler images to avoid redundant color regions affecting the model learning image features. For the present study, 1896 images (resolution of 975 × 674 × 3) were collected. Half of the ultrasound images displayed benign tumors, whereas the other half displayed malignant tumors. All images were labeled by gynecologists in accordance with the corresponding case and pathological report. The types of malignant tumors were including epithelial ovarian cancer (89.8%), germ cell tumor (5.4%), sex cord stromal tumor (3.4%), and sarcoma (1.4%).

#### Preprocessing

**Fig. 1:**
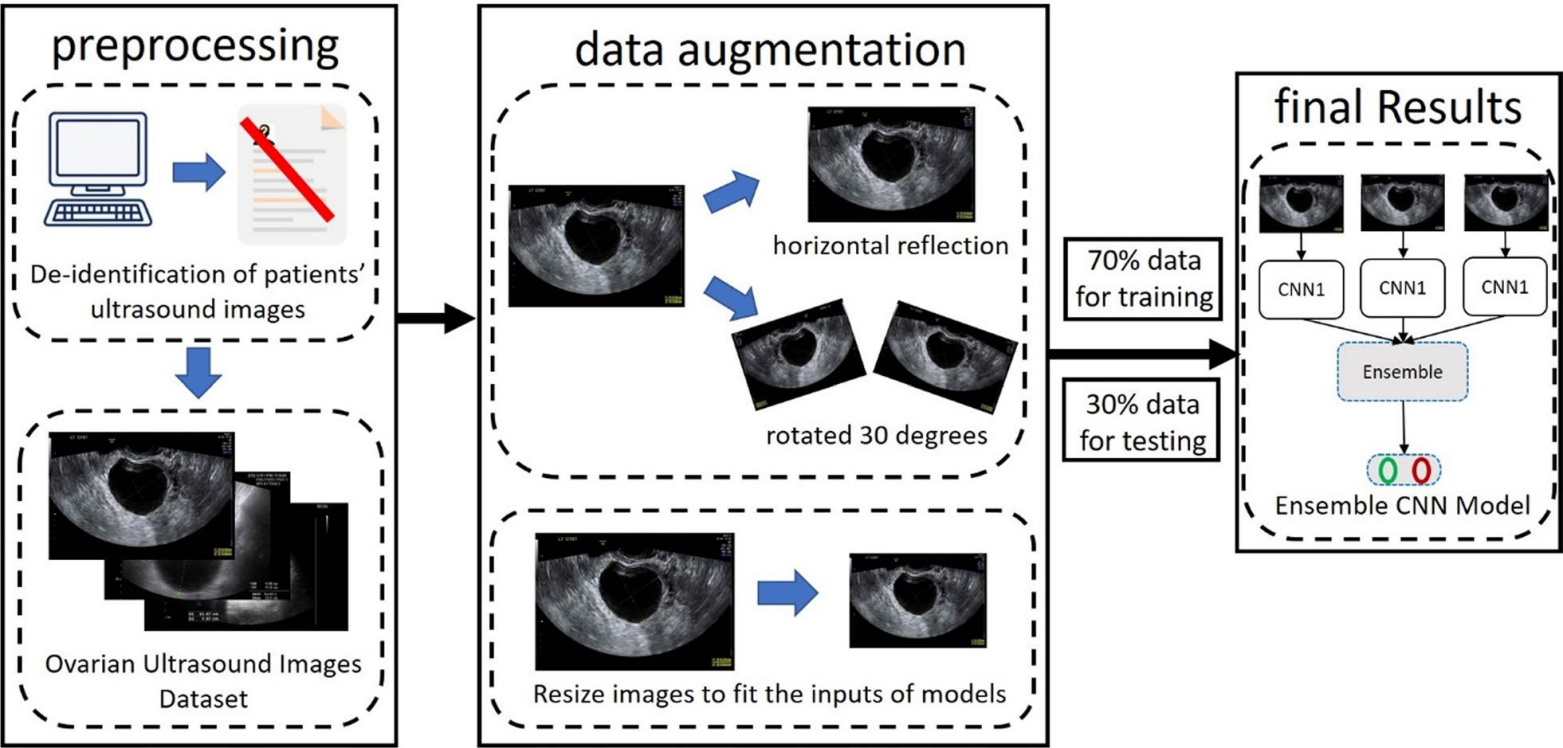
Training ovarian ultrasound image interpretation model

**Fig. 2:**
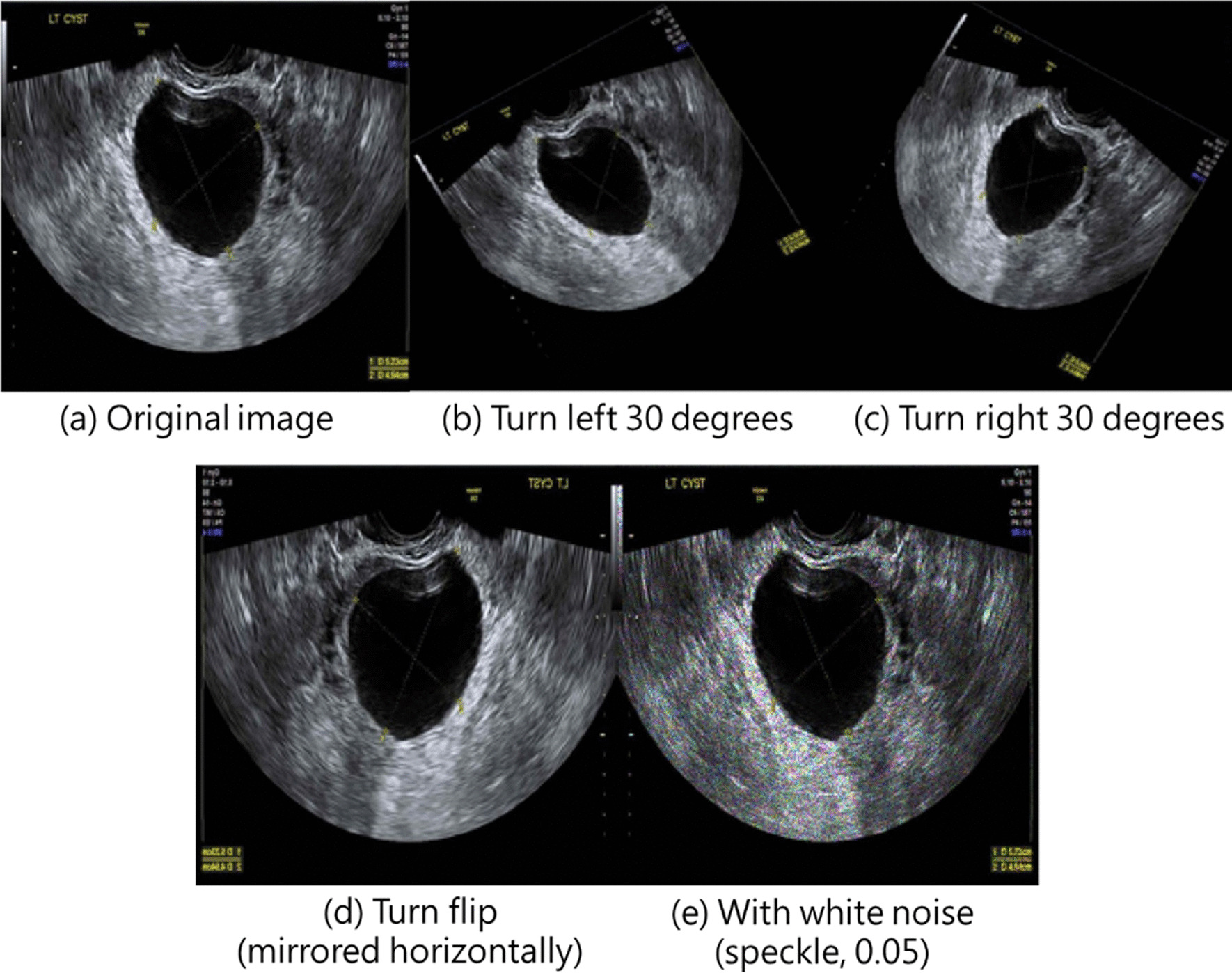
An example of the original image and its data augmentation. **a**–**e** present the original image, turn left 30 degrees, turn right 30 degrees, turn flip (mirrored horizontally), and with white noise (speckle, 0.05), respectively

The process from data preprocessing to model training is presented in Fig. 1. Medical record numbers and other relevant information were displayed on the raw images. To comply with IRB protocol, we removed medical record information from the images to ensure data confidentiality and avoid influencing subsequent model training and classification. From both benign and malignant tumor images, 70% were randomly selected and used as training datasets, and the remaining 30% were used as validation datasets. Training dataset images underwent data augmentation, which is a technique often used in machine learning to increase the diversity of training datasets, avoid model overfitting, and improve accuracy. In the present study, data augmentation involved randomized flipping (through which images were mirrored horizontally) and randomized rotation (through which images were rotated by 30° or − 30°). In addition, we add white noise (with speckle noise variance, 0.05) to each image in the training data to generate a number equal to the original training data, so the total number of images in the final training data set are 2654. Figure 2 shows an example of the original image and its data augmentation. Figure 2a–e present the original image, turn left 30 degrees, turn right 30 degrees, turn flip (mirrored horizontally), and with white noise (speckle, 0.05), respectively.

Ensemble learning uses multiple models, and all input images must be rescaled to fixed values to meet the requirements of each convolutional neural network (CNN). For example, the image input sizes for ResNet-50 and Xception are 224 × 224 × 3 and 299 × 299 × 3, respectively. After model training, the validation datasets were imported into a model for model assessment. The metrics used for evaluation were accuracy, sensitivity, and specificity. Random sampling and retraining of datasets were conducted after each assessment. Specifically, model accuracy, sensitivity, and specificity were tested ten times, and the mean of ten test results was set as the final assessment results.

##### CNN

In recent years, CNNs have achieved excellent results for tasks such as computer vision and image interpretation. A CNN is used to produce image feature maps and extract optimal features for training a network model for image classification. Numerous CNN architectures have been proposed, and their feature extraction methods can vary. GoogLeNet uses an inception network architecture, ResNet uses a residual learning framework, and DenseNet alleviates the vanishing-gradient problem and improves feature propagation. In the present study, ten prevalent CNNs (ResNet-18, ResNet-50, and ResNet-101 [28]; DenseNet-201 [29]; Inception v3 [30]; Darknet-19 and Darknet-53 [31]; ShuffleNet [32]; Xception [33]; and MobileNet-v2 [34]) were selected for classifying ultrasound images of benign and malignant ovarian tumors. Although CNN has excellent performance on many image-classification tasks, the excellent performance of CNN comes from classifying color images of common objects, people or animals in life, and we still do not ensure whether the same performance can also be achieved on ovarian ultrasound images. Therefore, we compared the ten aforementioned CNN architectures and used the CNNs as the foundation for constructing ensemble learning models, thereby allowing for the identification of the optimal model for classifying ovarian ultrasound images.

#### Ensemble learning

**Fig. 3:**
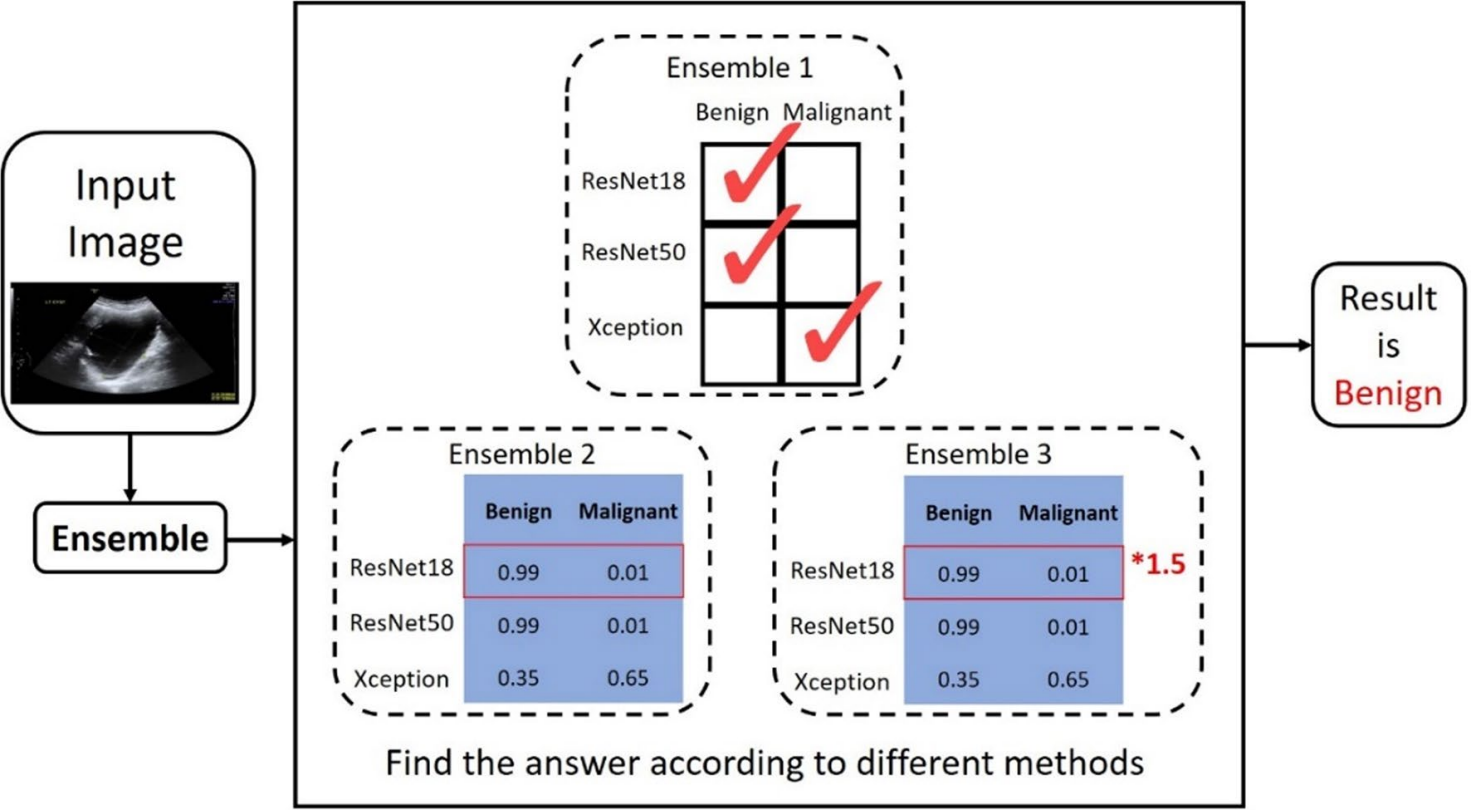
Three ensemble learning strategies. Ensemble 1 is based on majority voting, Ensemble 2 is based on sum of confidence scores, and Ensemble 3 is based on weighted sum of confidence scores

Ensemble learning combines the decisions of multiple supervised learning models to develop a more comprehensive perspective and improve the overall accuracy of predictions for a given problem. Common methods of ensemble learning include the aggregation of multiple weak classifiers and weighted voting. The only requirements for weak classifiers are more favorable performance relative to pure guessing and learnability. Weighted voting can help weak learners to achieve performance comparable to that of strong learners [35]. Ensemble learning is applicable in various areas such as augmented learning, feature selection, missing features, and data fusion [36]. Figure 3 is a schematic diagram of the three different ensemble methods used in the present study. Ensemble 1 involves majoritarian decision making with multiple models, that is, the model with the most votes wins, and the decision-making power of each model in Ensemble 1 is equivalent. Ensemble 2 involves decision-making with multiple models based on their confidence score. Each model rates the accuracy, sensitivity and specificity of image interpretation and gives a confidence score between 0 and 1, and the final interpretation results are defined by the sum of the maximum confidence score in each category. Ensemble 3 is similar to Ensemble 2, except that the confidence score of the model with the highest accuracy in each category is multiplied by 1.5, whereas the weights of the two remaining models remain unchanged. The final interpretation results are defined by the sum of the maximum confidence score in each category. Ensemble 3 is comparable to a scenario in which image interpretation is performed by three experts, of which one is more experienced and, thus, given more weight in decision making.

#### Effectiveness assessment

We used four indicators to evaluate the performance of the proposed method, namely overall accuracy (*ACC*), sensitivity (*SE*), specificity (*SP*), and standard deviation (*SD*), which are expressed using the equations as follows:

1 $${\varvec{A}}{\varvec{C}}{\varvec{C}}=\frac{TP+TN}{TP+TN+FP+FN}$$

2 $$SE=\frac{TP}{TP+FP}$$

3 $$SP=\frac{TN}{TN+FN}$$

4 $$SD=\sqrt{\frac{1}{N}\sum_{i=1}^{N}{({x}_{i}-\mu )}^{2}}$$

In the *ACC*, *SE*, and *SP* equations, *TP* denotes a true positive, *TN* denotes a true negative, *FP* denotes a false positive, and *FN* denotes a false negative. In the *SD* equation, *N* denotes the number of datasets, and *μ* is the mean of the overall data.

### Ensemble Grad-CAM

**Fig. 4:**
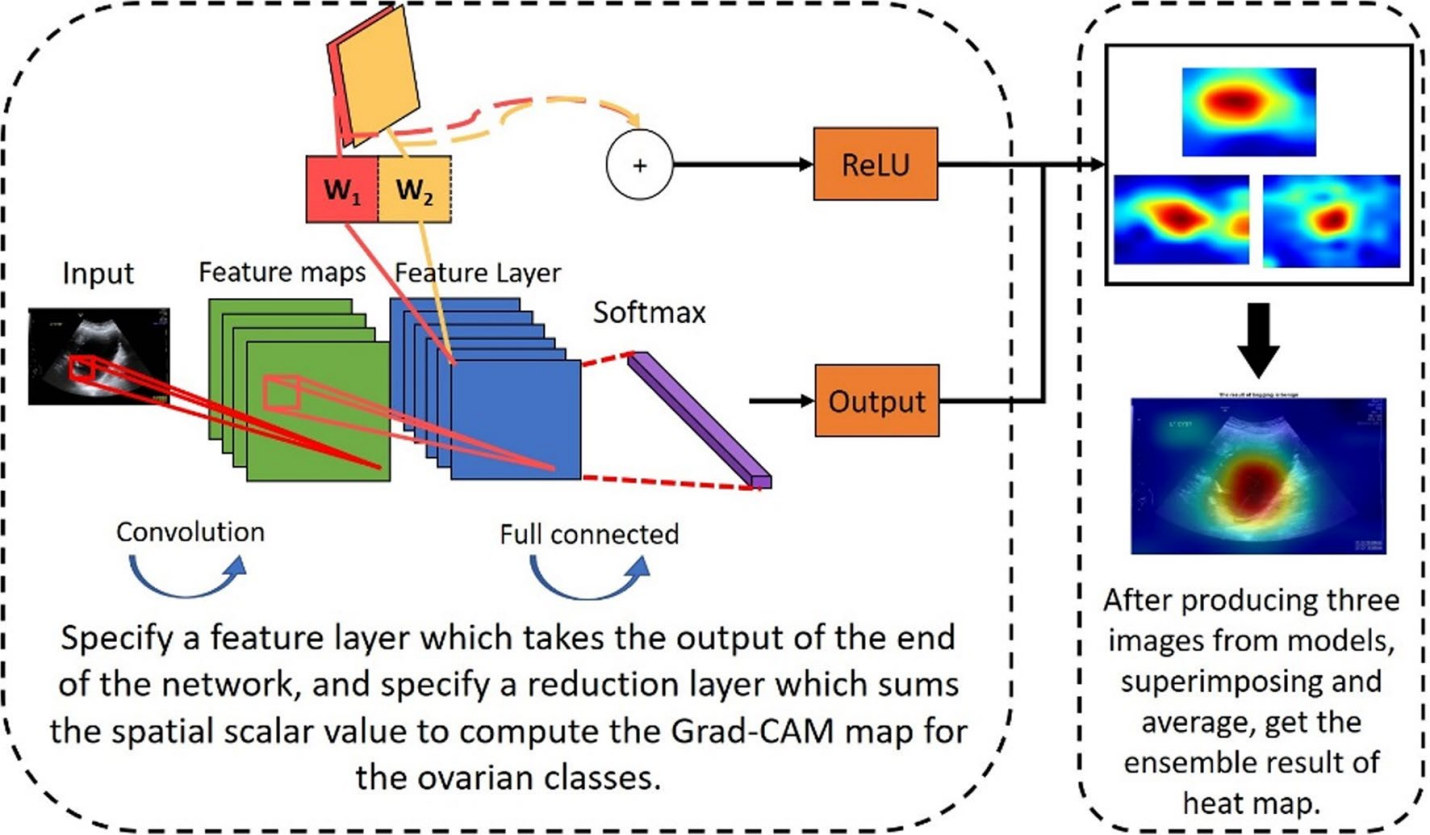
Ensemble Grad-CAM. Final heat map is produced by superimposing and averaging Grad-CAM heat maps

Although CNN provides excellent accuracy for various computer vision tasks, the lack of interpretability in the basis of decision-making causes experts to distrust the model’s decision and considerably increases the risk of misdiagnoses. Therefore, enabling experts to understand the decision-making basis of CNN models is crucial. Grad-CAM is proposed as a method for visualizing the decision of CNN models; this is achieved by obtaining a final heat map through the extraction of the feature layer at the end of a network model and performing a weighted sum of all feature maps. The red areas of a heat map are crucial references in the decision making of models. Although Grad-CAM can visualize the decision making of a model for use as a reference for experts, we found that the areas that the model's decisions focus on are sometimes inconsistent with t the expert. We still discovered that some confusing decisions such as the area of interest is on the background or without focusing tumors. And we also discovered that for a given ultrasound image and under the premise of accurate model classification, not all the red areas of heat maps aligned with the lesion that identified by experts. Consequently, the experts questioned the reliability of the model decisions. To resolve this problem, we also applied the concept of ensemble learning to Grad-CAM. Figure 4 is the flow chart of the proposed ensemble Grad-CAM. The heat maps produced by each model through Grad-CAM were superimposed and averaged to generate the final heat maps. This method can reduce the risk of models misidentifying non-lesion sites as the basis for decision making.

## Result

### Experiment setups

There are three main steps in our experiment: step (1) train and evaluate ten single CNN models; step (2) according to accuracy and computing costs, the top three trained CNN models in step (1) were selected to form three different ensemble CNN models; step (3) evaluate and compare the performances of the three different ensemble CNN models. The experiments were running on the PyTorch toolbox using Python programming language. Meanwhile, the software packages used in the experiment include Anaconda 3, PyTorch 1.6, CUDA10 and cudnn 8.0. The hardware platform is running on the computer with Intel CPU i9-7980XE, RTX 2080ti GPU with 11G memory, and 128G RAM. In the training process, the number of default epochs is set to 30 and the batch size was set to 32 in all experiments. The initial learning rate of optimizer using adaptive moment estimation (Adam) is set to 1e-4.

### Single network model performance

Table 1 presents the results obtained from ten CNN model architectures; specifically, we repeated the random sampling of training and validation data ten times and averaged the ten sets of results to obtain the final results. For *ACC*, *SE*, and *SP* performance, ResNet-50 had the highest *ACC* (90.51%) and *SP* (92%), and DenseNet-201 had the highest *SE* (89.77%); MobileNet-v2 had the poorest performance. The main reason we used the ten different CNNs was comparing which is the most suitable CNN architecture for the ultrasound imaging of ovarian tumor classification. For example, the DenseNet-201 model has a better performance than other selected CNNs by testing ImageNet dataset (https://www.image-net.org/). But the DenseNet-201 model is not necessarily the best CNN model for the ultrasound imaging of ovarian tumor classification. In other words, the more complex network architecture does not necessarily have better accuracy in every type of image. Furthermore, the ultrasound imaging of ovarian is a special form of image which was often widely applied in the fields of rapid tumor screening. Therefore, it is very important to find out which CNN architecture or components are suitable for the ultrasound imaging of ovarian tumor classification. According to our experimental results, ResNet50 has the highest accuracy for the ultrasound imaging of ovarian tumor classification. In addition to accurate interpretation results, the time required for interpretation should also be considered. Table 2 presents the time taken by each model to generate results from the input ultrasonic image data. According to Table 2, ResNet-18 took the least time (0.32 s) to produce results. Because of its high accuracy and speed, ResNet-18 was determined to be the optimal ensemble learning model.

**Table 1:** Results (mean ± standard deviation) of ten CNN model architectures after ten averages (from high to low accuracy)

CNN models	ResNet-50	ResNet-101	ResNet-18	DenseNet-201	Inception-V3
*ACC* (%)	**90.51 ± 4.36**	90.15 ± 3.03	89.90 ± 3.30	89.79 ± 2.84	89.79 ± 3.70
*SE* (%)	89.02 ± 5.44	89.65 ± 3.86	89.05 ± 5.27	**89.77 ± 4.16**	89.50 ± 4.11
*SP* (%)	**92.00 ± 5.95**	90.67 ± 3.86	90.77 ± 4.66	89.79 ± 4.10	90.06 ± 4.99

CNN models	Darknet-53	ShuffleNet	Xception	Darknet-19	MobileNet-v2
*ACC* (%)	89.53 ± 1.80	89.40 ± 3.78	88.79 ± 1.21	88.28 ± 1.90	87.41 ± 1.83
*SE* (%)	87.85 ± 3.99	87.43 ± 4.41	88.64 ± 2.95	88.05 ± 3.53	86.85 ± 1.98
*SP* (%)	91.20 ± 3.16	91.35 ± 3.89	88.93 ± 3.47	88.46 ± 3.36	87.99 ± 2.88

**Table 2:** Time (unit: s) taken by each model to generate interpretation results from input of an ultrasonic image (from least to most time taken)

CNN models	ResNet-18	Xception	ResNet-50	ShuffleNet	Darknet-19
Time (s)	0.32	0.67	0.81	0.83	0.97

CNN models	MobileNet-v2	Darknet-53	ResNet-101	Inception-v3	DenseNet-201
Time (s)	1.21	1.27	1.58	1.90	1.96

**Fig. 5:**
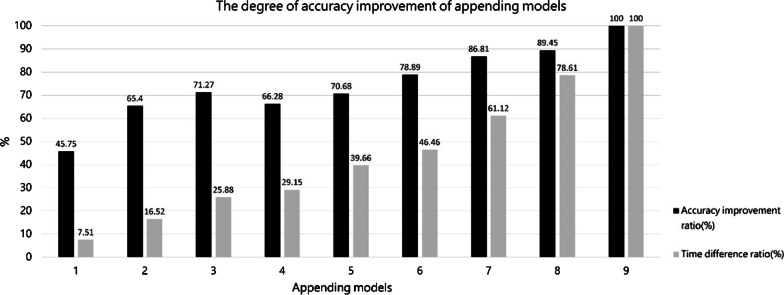
Relationship between accuracy improvement ratio and time difference ratio when models are appended to existing ResNet18 according to the orders in Table 2

In the order presented in Table 2, the models were gradually appended to the existing ResNet18 to assess the accuracy improvement of the ensemble models and the additional time required for ensemble models to interpret images. Figure 5 presents the association of the number of appended models with the accuracy improvement ratio and time difference ratio. With a range from ten ensemble models (maximum accuracy and maximum time consumption) to only ResNet-18 (minimum accuracy and minimum time consumption), the present study calculated the accuracy improvement ratios and time difference ratio following the increased number of appended models.

Figure 5 indicates a decrease in *ACC* (rather than an increase) after the four models were appended. *ACC* increased by 7.62% and time spent on interpretation increased by 17.31% when the number of appended models increased from three to six. In addition, whether the decision heatmap of the model is consistent with the expert is also the main reason why the model is considered to be used in ensemble learning. Therefore, we decided to use three ensemble models to achieve the benefits of high accuracy and fast processing. Finally, an ensemble network model was formed using ResNet-18, ResNet-50, and Xception for final ensemble learning.

### Performance of ensemble network models

Table 3 presents the *ACC*, *SE*, and *SP* results of the three ensemble learning methods based on three models (i.e., ResNet-18, ResNet-50, and Xception) after ten averages. Although Ensemble 3, which uses the weighted sum of confidence scores, had the highest *ACC*, the model’s sensitivity was reduced. Given that malignant tumors are more harmful than benign tumors and in consideration of clinical applicability, we aimed to reduce the risk of misdiagnosing malignant tumors. Table 3 indicates that Ensemble 2, which uses the unweighted sum of confidence scores, was only 0.04% less accurate (*ACC*) but also 0.41% more sensitive (*SE*) than Ensemble 3. For clinical applicability, we recommend Ensemble 2, whose *ACC*, *SE*, and *SP* were 92.15%, 91.37%, and 92.92%, respectively. Moreover, we perform pair sample *t*-tests on the ten test results of the three ensemble methods and the results of the best single CNN model (ResNet-50) to compare whether there is a statistical difference. The software tool we used to analyze the statistics was MATLAB® 2022a. The results show that there is no statistical difference (*p*-value: 0.061) between the Ensemble 1 and ResNet-50. The Ensemble 2 and Ensemble 3 both have statistical differences (*p*-value: 0.029 and 0.023, respectively) with ResNet-50. Such results indicate that Ensemble 2 and Ensemble 3 significantly outperform ResNet-50. In addition, the inference times of these three ensemble models for an ultrasonic image are 1.77 s., 1.79 s., and 1.8 s., respectively. This means that the computational cost required by the three ensemble methods is almost indistinguishable.

**Table 3:** Accuracy (ACC), sensitivity (SE), and specificity (SP) of Ensembles 1, 2, and 3 after ten averages

Ensemble methods	*ACC* (%)	*SE* (%)	*SP* (%)
Ensemble 1	92.01 ± 2.74	91.13 ± 3.45	92.88 ± 3.95
Ensemble 2	92.15 ± 2.84	**91.37 ± 3.60**	92.92 ± 4.00
Ensemble 3	**92.19 ± 2.88**	90.96 ± 3.96	**93.19 ± 4.19**

### Ensemble Grad-Cam result

**Fig. 6:**
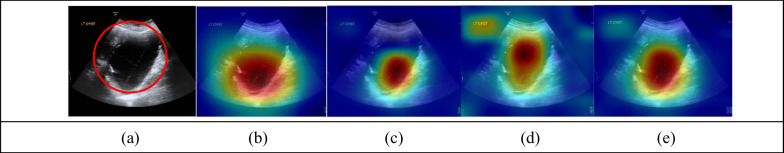
Comparison of expert interpretations with heat maps of each model. **a** Red circle is lesion site identified by experts. **b**–**d** Areas that drew the most attention from ResNet-18, ResNet-50, and Xception. **e** Heap map generated by superimposing and averaging heat maps of three models; its area of attention is most closely aligned with that of expert interpretations

After obtaining the model interpretation results, we used Grad-CAM to generate heat maps and invited experts to discuss with us the acceptability of the models’ explanations. Figure 6 presents an example of images that were compared and discussed. The red circle in Fig. 6a is the lesion site identified by experts. Figure 6b–d represent the areas that drew the most attention from ResNet-18, ResNet-50, and Xception. Although both model and expert interpretations classified the tumor as benign, ResNet-18’s area of attention (see Fig. 6b) was the closest to that of expert interpretations. By contrast, Xception’s area of attention (Fig. 6d) was partially out of focus, and it was partially focused on the background and text; therefore, Xception’s interpretation lacked persuasiveness. We decided to combine the perspectives of all three models by superimposing and averaging their heat maps, thereby creating Fig. 6e. As indicated by Fig. 6e, the degree of attention paid to the background and ovarian tumor decreased and increased, respectively. In Fig. 6e, the area of attention was closest to that of expert interpretations. The heat map produced by superimposing and averaging the three model heat maps aligned more closely with the discussion results of the three experts.

## Discussion

A model’s confidence score for decision making determines whether experts adopt or accept its decision. Usually, a higher confidence score (rated by the model) is associated with a lower risk of interpretation errors and higher level of trust among experts in a model’s decisions. For clinical applications, artificial intelligence (AI) is a method for conducting auxiliary diagnosis. An AI-based diagnostic system requires the development of an optimal threshold for the confidence score of decision-making; this allows clinical staff to determine when they can trust the auxiliary diagnostic system and when they should reinterpret the results manually. An excessively high threshold indicates that more data are required for manual reinterpretation by clinical staff, which defeats the purpose of using the auxiliary diagnostic system to reduce labor and time cost. An excessively low threshold indicates an increased risk of incorrect decisions being made, which reduces the confidence of clinical staff in the system’s decisions; they may even reject system-derived results to avoid misdiagnoses and disputes.

**Fig. 7:**
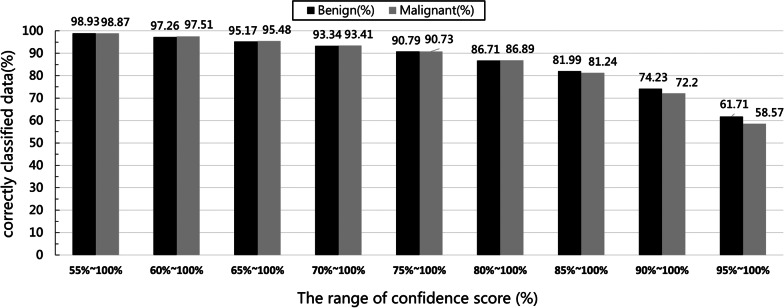
Percentage of accurately interpreted benign and malignant tumors by confidence score threshold

To further explore the relationship between the range of confidence scores for decision-making and the amount of accurately interpreted data, we calculated the amount of benign and malignant tumor data that were distinguished in accurate interpretations under various confidence score thresholds. The results are presented in Fig. 7. When the confidence score threshold was set to  ≥ 95%, 61.71% and 58.57% of benign and malignant tumor images, respectively, were included. That is, among the accurately interpreted data, nearly 40% of the images required manual reinterpretation when the confidence score was set to between 95 and 100%; this defeats the purpose of using the auxiliary diagnostic system to reduce labor and time cost.

When the confidence score threshold was set to between 80 and 100%,  ≥ 86% of images were included regardless of tumor type. That is, among the accurately interpreted data, less than 14% of the images had a confidence score of between 50 and 80%. A confidence score threshold of between 80 and 100% can lead to a more balanced relationship between the risk of interpretation errors and the deployment of medical resources or clinical personnel to reinterpret images.

To obtain the optimal threshold value of a confidence score, we applied the analysis method as follows. Images were excluded when a confidence score did not exceed the threshold value, and the overall accuracy of the included images was calculated. Starting from 80%, we gradually increased the threshold value by increments of 1% to observe the effects of threshold changes on overall interpretation accuracy and determine the amount of data requiring manual reinterpretation. The analysis results indicated that a higher threshold value could improve accuracy, but they also revealed an increase in the amount of data requiring manual reinterpretation. When the confidence score was set to 99%, an overall accuracy of 99.38% was obtained following image exclusion, but the excluded data accounted for 61.75% of the total data.

Through discussions with clinical experts, we discovered that the optimal results were achieved by setting the confidence score to 86%. Because according to the statistics of clinical experts, there is an average about 20% inconsistency between the interpretation results of the experts based on the ultrasound images and the results of the final tumor section pathology report, so we set the images with confidence scores more than 86% (accounting for 80% of all testing data) can directly adopt the decision of the model. In other words, there will be 20% of the testing data and we will recommend that clinical experts make manual judgment again to confirm the final result. Among the 568 images that served as the validation data for the present study, 112 images (approximately 20% of the total validation data) were excluded for a confidence score of less than 86% and for images requiring manual reinterpretation. In the remaining images, 446 images were correctly identified and other 10 images were incorrectly identified, indicating an overall accuracy rate of 97.78%. If no threshold was set, 45 images would be inaccurately interpreted, which is equivalent to an overall accuracy rate of 92.1%.

In order to compare the consistency between expert’s image classified results and the pathology report of tumor section, we asked a clinical expert to classify all testing ultrasound images for benign and malignant. The clinical expert is a gynecologist with 21 years of experience and about 2500 patients’ ultrasound interpretations per year. The results were showed in Table 4, the *ACC* of the proposed method was also added. The results of the comparison show that the accuracy of the proposed method is at least 12% higher than that of the expert. Such results mean that the accuracy of our proposed method has exceeded the level of experts.

**Table 4:** Comparison of the pathology report (ground truth) with expert scoring and our proposed method

Ground truth/manual or automatic classification	*ACC* (%)
The pathology report/expert scoring	79.63 ± 4.45
The pathology report/proposed method	**92.15 ± 2.84**

Table 5 presents the comparison of the proposed method with other methods in terms of the interpretation of ovarian ultrasound images. A study [37] used the k-nearest neighbors method to classify 2600 ultrasound images of benign and malignant tumors and reported an accuracy of 100%. However, the aforementioned study lacked data diversity because the images were collected from only 20 women, with each woman contributing 130 ultrasound images to the dataset. Because individual differences exist in biomedical data, the lack of diverse data for model training can lead to validation results having limited generalizability and low reference value in clinical settings. Another study [38] applied a traditional machine learning method (i.e., fuzzy forest) to classify 469 ultrasound images of benign and malignant tumors collected from 469 women; although the image dataset was diverse, the study only achieved an accuracy of 80.6%. CNN and transfer learning were applied in another study [39] to classify the tumors in 988 ovarian ultrasound images as benign, malignant, or unidentifiable; the study achieved an accuracy of 87.5%. In the present study, the proposed CNN-based method outperformed [39] in terms of the quantity and accuracy of its validation data.

**Table 5:** Comparison of proposed method and related literature

Method	Subject	Number of images	Classifier	Accuracy (%)
Rajendra et al. [37]	20 subjects age: 29 to 74	2600 (1300 benign/13000 malignant) 130 images from each subject	KNN	100%
Rajendra et al. [38]	469 subjects age: 23 to 90	469 (238 benign/231 malignant)	fuzzy forest	80.6%
Wu et al. [39]	988 subjects age: 7 to 84	988 (the number of images in each class were not provided)	CNN (DenseNet)	87.5%
Our proposed method	587 subjects age: 11 to 85	1896 (948 benign/948 malignant)	Ensemble CNN	92.15%

## Conclusion

In the present study, we proposed an automatic system that utilizes an ensemble CNN to interpret ovarian tumor ultrasonic images. The system incorporates technologies such as image preprocessing, data augmentation, and ensemble Grad-CAM. For our validation strategy, we repeated the random sampling of the training and validation data ten times to verify model robustness. The mean ACC, SE, and SP of the single network model with optimal performance were 90.51%, 89.02%, and 92%, respectively. The present study proposed the ensemble method on the basis of the confidence scores of multiple decision-making models, which achieved a mean ACC, SE, and SP of 92.15%, 91.37%, and 92.92%, respectively. The proposed method increased the mean ACC, SE, and SP of the single network model with optimal performance by 1.64%, 2.35%, and 0.92%, respectively. From the comprehensive perspective of data quantity, data diversity, robustness of validation strategy, and overall accuracy, the proposed method outperformed the methods used in other studies.

Future studies should explore the detection of tumors in ultrasound images. In real-life clinical practice, tumors are absent from most ultrasound images captured by ultrasound machines. By overcoming the challenge of determining the presence of ovarian tumors in an image we can accelerate the screening and preprocessing of images and reduce the risk of misdiagnoses. Furthermore, no study has attempted to distinguish between abdominal and vaginal ultrasound images during data concentration. The features of abdominal and vaginal ultrasound images are different. Therefore, misdiagnosis rates can be reduced by training a model to capture multiple sites in ultrasound images. We will also continue to add more patient images to increase data diversity. A comprehensive dataset can produce a more robust clinical applicability with respect to the incorporation of AI into clinical applications.

## Data Availability

The datasets generated and/or analysed during the current study are not publicly available due to patients’ privacy and research permissions but are available from the corresponding author on reasonable request.
